# Protective Effect of Oral BCG and Inactivated *Mycobacterium bovis* Vaccines in European Badgers (*Meles meles*) Experimentally Infected With *M. bovis*

**DOI:** 10.3389/fvets.2020.00041

**Published:** 2020-02-04

**Authors:** Ana Balseiro, José Miguel Prieto, Vega Álvarez, Sandrine Lesellier, Dipesh Davé, Francisco J. Salguero, Iker A. Sevilla, José Antonio Infantes-Lorenzo, Joseba M. Garrido, Hans Adriaensen, Ramón A. Juste, Marta Barral

**Affiliations:** ^1^Departamento de Sanidad Animal, Facultad de Veterinaria, Universidad de León, León, Spain; ^2^Servicio Regional de Investigación y Desarrollo Agroalimentario (SERIDA), Centro de Biotecnología Animal, Gijón, Spain; ^3^Animalien Osasuna, NEIKER-Instituto Vasco de Investigación y Desarrollo Agrario, Derio, Spain; ^4^Anses Nancy Laboratory for Rabies and Wildlife, Malzéville, France; ^5^Bacteriology Department, Animal and Plant Health Agency, Addlestone, United Kingdom; ^6^Public Health England, Porton Down, Salisbury, United Kingdom; ^7^VISAVET Health Surveillance Centre, Universidad Complutense de Madrid, Madrid, Spain; ^8^CIRE Plateform, Service Imagerie, UMR PRC, Centre INRAE Val-de-Loire, Nouzilly, France

**Keywords:** tuberculosis, badger, BCG vaccine, *Mycobacterium bovis* heat-inactivated (HIMB) vaccine, efficacy

## Abstract

In Europe, badgers (*Meles meles*) are recognized as major tuberculosis (TB) reservoir hosts with the potential to transmit infection to associated cattle herds. Recent studies in Spain have demonstrated that vaccination with a heat-inactivated *Mycobacterium bovis* vaccine (HIMB) successfully protects captive wild boar and red deer against progressive disease. The aim of this study was to evaluate the efficacy of two oral vaccines against TB in a badger model: the live-attenuated *M. bovis* bacillus Calmette-Guérin BCG vaccine (Danish strain) and a HIMB vaccine. Twenty-four badgers were separated in three treatment groups: oral vaccinated with live BCG (10^8^ CFU, *n* = 5), oral vaccinated with HIMB (10^7^ CFU*, n* = 7), and unvaccinated controls (*n* = 12). All badgers were experimentally infected with *M. bovis* (10^3^ CFU) by the endobronchial route targeting the right middle lung lobe. Throughout the study, clinical, immunological, pathological, and bacteriological parameters of infection were measured. Both vaccines conferred protection against experimental TB in badger, as measured by a reduction of the severity and lesion volumes. Based on these data, HIMB vaccination appears to be a promising TB oral vaccine candidate for badgers in endemic countries.

## Introduction

Animal tuberculosis (TB), caused by infection with members of the *Mycobacterium tuberculosis* complex (MTBC) (mainly *M. bovis*, and to a lesser extent, *M. caprae*), is a major economic disease of livestock worldwide that can also cause zoonotic TB in humans (1). In spite of major efforts invested in the control of the disease in cattle, its major domestic reservoir, TB is still present in many European countries. A TB outbreak in livestock may occur due to persistence of the pathogenic mycobacteria within the herd (i.e., residual and environment infection) or due to the introduction into a previously TB-free herd (2). Wildlife hosts are also susceptible to *M. bovis* and can act as a reservoir for the infection for livestock. In Europe, in the United Kingdom (UK) and Republic of Ireland (ROI), badgers (*Meles meles*) are recognized as major TB reservoir hosts with the potential to transmit infection to associated cattle herds (3, 4), while in Spain the wildlife species most commonly associated with outbreaks in domestic animals is the wild boar (5). Vaccination of badgers has been proposed as a long-term control strategy for the disease in the UK and Ireland (6–8) and experimental studies have demonstrated that vaccination with live attenuated bacillus Calmette-Guérin (BCG) vaccine is protective in badgers, and also in wild boar (9–15). However, the use of a live vaccine (BCG) at protective doses in badger is only licensed in the UK by the intramuscular route and its use in baits is controversial (16). That is because there are particular challenges associated with the development of a live oral BCG vaccine, not least maintaining the survival of BCG in baits until deployment in the field, but also with issues arising from release of a live vaccine into the environment. In this sense inactivated vaccines are attractive for field delivery because they are expected to be more stable in baits, especially under high environmental temperatures, and safer in the field conditions as it is a vaccine based on the dead bacteria (17). Recent studies in Spain have begun to address these issues by demonstrating that oral vaccination with a heat-inactivated *Mycobacterium bovis* vaccine (HIMB) successfully protects captive wild boar (18) and red deer (19) against progressive disease. Field studies have also demonstrated the efficacy of oral HIMB vaccination of piglets against TB in endemic free-ranging wild boar populations (17).

The aim of this study was to evaluate the efficacy of two oral vaccines against TB in captive badgers: the live-attenuated *M. bovis* BCG vaccine (Danish strain) and a heat-inactivated *M. bovis* (HIMB) vaccine.

## Materials and Methods

### Ethical Statement

All methods were carried out in accordance with relevant guidelines and regulations. All experimental protocols were approved by ethical committees from Government of Principality of Asturias and Government of Basque Country with license reference numbers: PROAE 20/2015 and NEIKER-OEBA-2015-009.

### Badgers and Experimental Design

Twenty-four badgers (13 males and 11 females) were trapped between March and September 2016 and 2017 from a cattle TB-free area in Spain (Nava, Asturias), 43°21'30”N, 5°30'20”W. Badgers were tested for avian and bovine TB by Interferon-Gamma (IFN-γ) Release Assay (IGRA) enzyme linked immunosorbent assay (ELISA) and IGRA ELISPOT before the start of the study in order to verify the negative pre-sensitization of the animals to mycobacteria. All the badgers were housed in an outdoor purpose-built facility in Servicio Regional de Investigación y Desarrollo Agroalimentario (SERIDA) in stable social groups. Pens were consolidated with underground wired sheet covered with soil amenable to digging and with permanent access to large wooden sett with internal straw bedding, water (for drinking and bathing) and various environmental enrichment features (e.g., wooden structures and branches). Food was provided daily, and the welfare of the animals was monitored daily. All animals remained in good clinical condition throughout this part of the study.

All the procedures in badgers were conducted under general anesthesia induced by a cocktail of butorphanol, medetomidin, and ketamine as previously conducted (20). Badgers were identified using a subcutaneous microchip placed in the left side of the neck and randomly allocated in three treatment groups: oral vaccinated with live Danish BCG [10^8^ colony forming units (CFU), *n* = 5], oral vaccinated with HIMB (10^7^ CFU*, n* = 7), and unvaccinated controls (*n* = 12). The vaccination with BCG and HIMB was conducted in the outdoor facility at SERIDA. The vaccines were delivered orally under general anesthesia by dispensing 200 μl vaccine solution in PBS (using disposable pastettes directly on the tonsils of animals). The control badgers were also anesthetized on the day of vaccination for blood sample collection but did not receive a sham vaccination. Eleven weeks after vaccination, all badgers were moved to Instituto Vasco de Investigación y Desarrollo Agrario (NEIKER) into their wooden setts and housed in biosafety level three containment facilities (one large room for each vaccine treatment group and two for the controls) for acclimatization for 2 weeks prior to experimental challenge with pathogenic *M. bovis*. All 24 badgers were experimentally challenged with *M. bovis* by the endobronchial route at week 13. Throughout the study, clinical, immunological, pathological, and bacteriological parameters of infection were measured. Rectal temperature and body weight were measured before and after challenge and at every sampling. Blood samples were collected at vaccination (week 0), 3 weeks post-vaccination (PV), 8 weeks PV, 13 weeks PV (challenge-infection), 2 weeks post-infection (PI), 7 weeks PI, and 12 weeks PI from the jugular vein in both serum tubes and heparinized blood tubes (Vacutainer®, BD Diagnostics, Plymouth, UK). Urine was obtained by bladder massage and tonsillar, nasal, and fecal swabs were collected during the samplings after-challenge (weeks 2, 7, and 12 PI). Tracheal swabs and feces were collected after post-mortem examination. Euthanasia was conducted 12 PI by injecting a lethal dose of intravenous barbiturates (Dolethal®, pentobarbital sodium).

### Immunology on Blood Samples

#### Cellular Assays

##### Whole blood IGRA ELISA

IGRA was performed as previously described (21); 1.5 ml aliquots of heparinized blood diluted 1:1 in RPMI medium (Fisher, Hampton, USA) supplemented with 1% sodium heparin (Roche, Basilea, Switzerland) and 1% penicillin/streptomycin (pen/strep) (Fisher, Hampton, USA) was stimulated with a final concentration of 30 μg/ml bovine and avium tuberculins (Spanish PPD-B and PPD-A, respectively, CZ Vaccines, Porriño, Spain), with pokeweed mitogen at a final concentration of 5 μg/ml (Sigma-Aldrich, St. Louis, USA), with CFP-10/ESAT-6 (Lionex, Braunschweig, Germany) at a final concentration of 5 μg/ml protein cocktail or without antigen (RPMI+Pen/strep), and kept at 37°C with 5% CO_2_ for a minimum of 16 h. Post-incubation, supernatants were collected in duplicate aliquots (250 μl) and stored at −80°C. IFN-γ levels were assessed by sandwich ELISA using anti-badger IFN-γ capture monoclonal antibody 10H6-C1 and biotinylated monoclonal antibody 11B9 (Animal and Plant Health Agency, UK). The cut-off point of 0.044 (using combined PPD-B and PPD-A values) was used to identify positivity to *M. bovis* infection.

##### IGRA ELISPOT

A direct ELISPOT assay was also conducted as previously described (22). Fresh isolated peripheral blood mononuclear cells (PBMCs) (2 × 10^5^ cells per duplicated wells) diluted in RPMI complete medium made up of 5% fetal calf serum (Sigma-Aldrich, St. Louis, USA), 1% pen/strep, 1% non-essential amino acid (Gibco, Hampton, USA), and 0.1% mercaptoethanol (Fisher, Hampton, USA) were used. PBMCs were stimulated with Spanish PPD-B and PPD-A at a final concentration of 30 μg/ml, mitogen Concanavalin A (Sigma-Aldrich, St. Louis, USA), antigen 85A (a secretory protein of *M. tuberculosis* and BCG) and antigens CFP-10/ESAT-6 at final concentrations of 5 μg/ml or no antigen (RPMI complete) for 16–20 h in wells pre-coated with monoclonal antibody 10H6-C1(10 mg/ml) in carbonate buffer. The IFN-γ producing cells were detected with biotinylated monoclonal antibody 11B9. The ELISPOT results were expressed as number of spot forming unit/million cells.

##### PBMC IGRA ELISA

Isolated PBMCs were also used for IGRA ELISA. A volume of 700 ul PBMCs were stimulated with 700 ul of Spanish PPD-B and PPD-A at a final concentration of 30 μg/ml, mitogen Concanavalin A, and antigens CFP-10/ESAT-6 at final concentrations of 5 μg/ml or no antigen (RPMI complete). Plates (48 wells) were kept at 37°C with 5% CO_2_ for a minimum of 20–24 h. Afterwards they were centrifuged at 800 g for 10 min. Supernatants (250 μL) were taken in duplicate and frozen at −80°C. An IGRA previously described for stimulated whole blood (see above) was carried out and analyzed using PPD-B and PPD-A values with a cut-off of 0.044 nm (21).

#### Serological Assay

##### P22 ELISA

Serum samples were tested by an in-house indirect ELISA to detect antibodies against the *M. bovis* P22 complex, following the protocol described previously by Infantes-Lorenzo et al. (23). Briefly, plates were coated with P22 at 10 μg/ml overnight in phosphate buffer saline (PBS) and then blocked with 5% skimmed milk powder solution (prepared in PBS). After three washes with PBS containing 0.05% Tween-20 (PBST), sera were added to duplicate wells as a 1:100 dilution in PBS-skim milk and incubated for 60 min. Horseradish peroxidase-conjugated CF2/HRPo anti-badger IgG (100 μl) (Animal and Plant Health Agency, UK) (24) was diluted to 1.5 μg/ml in PBS and added to the plates. Then plates were incubated with 3,3′,5,5′-tetramethylbenzidine substrate (Perbio, Skane Lan, Sweden) for 15 min in the dark at room temperature. The reaction was stopped by adding 100 μl of 2 M H2SO4. Optical density (OD) was measured at 450 nm using an ELISA reader. Negative control serum samples from UK tuberculosis-free captive badgers were included in every plate in quadruplicate. Positive controls were obtained from UK badgers experimentally infected with *M. bovis*. Sample results were expressed as an ELISA percentage E%, calculated using the following formula:

E% = mean sample OD / (2 × mean of negative control OD) ×100%. The cut-off point was set up in previous work (23). Serum samples with E% values >120 were considered positive.

### Preparation of *M. bovis* Bacillus Calmette-Guérin (BCG) Vaccine

The *M. bovis* BCG inoculum was prepared as previously described (14). Briefly *M. bovis* BCG Danish 1331 strain (ATCC, Ref. 35733^TM^) was prepared by the Statens Serum Institute (Copenhagen, Denmark) to reach 10^8^ colony forming units (CFU)/ml in monosodium glutamate. It was purchased by APHA.

### Heat-Inactivated *Mycobacterium bovis* Vaccine (HIMB)

HIMB was provided by NEIKER. The strain, first isolated from a naturally infected wild boar on Coletsos medium, was propagated in Middlebrook 7H9 broth enriched with oleic acid-albumin-dextrose-catalase (OADC Enrichment; Difco, USA) for 2–3 weeks (18). Inactivation of bacteria was carried out in an airtight bottle submerged in a water bath at 84–85°C for 45 min (25). The concentration before inactivation was 5 x 10^7^ CFU/ml.

### *Mycobacterium bovis* Challenge Strain

A Spanish *M. bovis* field strain (SB0339) isolated from a tuberculous wild boar was diluted to ~10^3^ CFU/ml in PBS and loaded in luer lock syringes (3 ml capacity). At challenge, the syringes were vortexed, and the challenge inoculum (1 ml) immediately instilled through the internal catheter of a fibroscope (Olympus URF P2 3.6 mm × 70 cm, 1.8 mm channel), to the bronchial entrance of the right middle lung lobe of anesthetized badgers positioned in ventral recumbency. Anesthetized badgers were then placed on their right side until recovery from general anesthesia, to reduce the risk of the inoculum draining to the left lung through the bronchial bifurcation.

### Post-mortem Examination

A detailed post-mortem examination was conducted of 26 fresh tissues following standard protocols (13–15). Collected tissues included tonsils, salivary glands, left, and right parotid lymph nodes (LNs), left and right mandibular LNs, left and right retro-pharyngeal LNs, anterior and posterior mediastinal LNs, left and right bronchial LNs, lung lobes, mediastinum, left and right axillary LNs, heart, left and right inguinal LN chains, left and right popliteal LNs, spleen, hepatic LN, mesenteric LN, liver, left and right kidneys and heart (14). The severity (from 1 to 4) of the visible lesions was recorded by veterinary pathologists blinded to badger treatment groups. In order to calculate the overall disease burden score (DBS), tissues were also collected for culture (stored frozen at −20°C), and for histological examination (in 10% buffered formalin). New sets of sterile instruments were used between tissues to avoid bacterial cross-contamination. The fixed lungs were inflated with 10% buffered formalin for further magnetic resonance imaging (MRI) scanning and subsequent histological analysis before careful direct examination for visible lesions. The lesions scores were calculated for each tissue as previously described (14). Lungs were sliced at 0.5 cm in order to find TB compatible lesions in the parenchyma. Total lung scores were then calculated by adding the lesion scores for all lung lobes with lesions.

### Magnetic Resonance Imaging (MRI) Acquisition and Analysis

The formalin fixed lungs were scanned by an open MRI scanner (Hitachi, Aperto, Lucent) 0.4T (Tesla) endoted at magnetic field gradients of 25 mT/m at Center de Recerca en Sanitat Animal (CRESA) (Barcelona, Spain). TB granulomas/lesions differ from the healthy parenchyma in the intensity level on the T_1_ (spin-lattice or longitudinal relaxation time) and T_2_ (spin-spin or transverse relaxation time) images. Thus, two separate MRI sequences were performed on each lung, so as to obtain two different image contrasts. The T_1_ 3D and T2 2D were, respectively, the RSSG (RF-Spoiled Steady-state Acquisition Rewound Gradient Echo) and the FSE (Fast Spin Echo). The acquisition parameters were set as follows for the: (i) T_1_ 3D: Repetition Time (TR) = 30 ms, Echo Time (TE) = 10 ms, flip angle = 40 degrees, Field-of-View (FOV): 200 × 200 mm^2^, matrix: 156 × 256^2^ and a slice thickness of 1.5 mm, resulting in a voxel size of 1.28 × 0.78 × 1.5 mm^3^. A bandwidth of 14.1 kHz/Px was used, 4 Number of Excitations (NEX) producing an acquisition time of 10 min 47 s; and (ii) T_2_ 2D: TR = 3,000 ms, TE = 100 ms, flip angle = 90 degrees, Echo Train Length (ETL): eight and bandwidth of 19.9 kHz/Px. The FOV was 200 × 200 mm^2^, matrix: 224 × 320^2^ and slice thickness: 2 mm, making a voxel size: 0.89 × 0.62 × 2 mm^3^. With 6 NEX, an acquisition time of 12 min 21 s was generated.

The analysis of the MRIs was done into two steps; they were first converted and afterwards underwent segmentation. In step number 1, the Digital Imaging and Communication in Medicine (DICOM) images were exported from the acquisition machine onto a PC analysis workstation. The DICOM images were converted to the Neuroimaging Informatics Technology Initiative (NIfTI). In step number 2, the NIfTI images were read with ITK Snap, which is generally used to segment 3D medical image structures. The segmentation was then done automatically with a Human-Machine Interface (HMI) created *in-situ*. This HMI created a bit mask by assigning 1 to voxels thresholded between a minimum and a maximum values and by assigning 0 to the rest of the image. These minimum and maximum were discriminated on the NIfTI images. The HMI superimposed directly the mask on the T_1_ NIfTI images in the ITK Snap software. The voxel under investigation were then colorized in red. It was therefore necessary to correct the automatic segmentation and to carry out a manual segmentation and consequently eliminate the voxels that did not correspond to lesions. To do this the T_2_ images were loaded simultaneously with the T_1_ images which allowed confirming the belonging of the dots to a specific class. Indeed, T_2_ offered a different contrast than T_1_ in order to confirm TB lesion images. Once the segmentation was satisfactory, it was possible to edit the volumetry that corresponded to the segmentation and thus to the lesions. All segmentations were afterwards blindly re-read through a third party for validation.

### Culture, Real Time-Polymerase Chain Reaction (RT-PCR), and Spoligotyping

Tissues were weighed and individually homogenized in 5–15 ml saline solution (0.85%) with a GentleMacs dissociator (Miltenyi Biotec, Madrid, Spain). Each homogenate was inoculated (100 μl per tube) in one BD BBL™ mycobacteria growth indicator tubes (MGIT™) (Becton Dickinson, Franklin Lakes, NJ, USA) supplemented with BACTEC™ MGIT™ growth supplement and PANTA™ antibiotic mixture according to manufacturer's instructions (Becton Dickinson, Franklin Lakes, NJ, USA). Afterwards they were plated on to six modified 7H11 agar plates (100 μl onto each plate) as previously reported (14). Three plates were made using BBL^TM^ 7H11 agar (Becton Dickinson, Franklin Lakes, NJ, USA) and the other three using Middlebrook 7H11 agar base (Sigma-Aldrich, St. Louis, MO, USA). The inoculated plates were incubated at 37°C for 12 weeks. Afterwards, CFU were counted and the total bacterial load was calculated, as the mean number of colonies on the 7H11 plates. In order to simplify and normalize for statistical analysis, an integer score ranging between 1 and 7 in a base 10 logarithm was assigned to the bacterial load. All the positives plates/tubes were confirmed as MTBC by multiplex PCR (26). Additionally, colonies from three positive cultures from each badger, obtained from LNs from the head, the thorax, and any other location outside the thorax were spoligotyped (27).

Clinical samples including tonsillar and nasal swabs from weeks 2, 7, and 12 PI, and tracheal swab obtained at necropsy, were individually homogenized in 1 ml saline solution (0.85%). The resulting suspensions were plated (100 μl per plate) onto six solid medium plates and inoculated (100 μl) in one MGIT. The same amount of urine (100 μl) was cultured in the same way as swab homogenates. Feces (2 gr) obtained at necropsy (12 weeks PI) and fecal swabs collected from weeks 2 and 7 PI, were homogenized in 38 ml or 15 ml saline solution (0.85%), respectively, and decontaminated with oxalic acid (5% final volume) before being inoculated in the same media (13). The inoculated plates were incubated at 37°C for 12 weeks and tubes introduced in a BACTEC™ MGIT™ 960 System for an incubation protocol of 42 days. Afterwards, CFU were counted and the total bacterial load was calculated, as the mean number of colonies on the 7H11 plates. For confirmation of all the positives cultures, DNA from isolates was submitted to a multiplex real-time PCR assay to identify MTBC, *M. avium* and the genus *Mycobacterium* according to the protocol previously described (26). Additionally, MTBC colonies from three positive cultures from each badger, representing isolates obtained from three different body sites (LNs from the head, the thorax and any other location outside the thorax) were spoligotyped (27).

### Histopathology

Collected samples were processed by standard protocols and assessed by histopathology—hematoxylin and eosin (HE) and Ziehl-Neelsen (ZN) staining specific for acid-fast bacilli (AFB). The histological scores were calculated for each tissue. Granulomas were classified based on cell composition, severity and number of mycobacteria from types 1 to 4 (14). Granuloma type 1 contained lymphocytes, epithelioid cells and plasma cells. Granuloma type 2 presented the same cellular types as granuloma 1 and a coagulative necrotic center; granuloma type 3 a caseous necrotic center and granuloma type 4, the most severe, a caseous and mineralized necrotic center. Tissue sections with active lymphoid follicles only were scored as 0 and were not included in the calculation of final scores. AFB abundance was scored from 0 to 2 (0 = none; 1 = scarce; 2 = abundant). The histological score was based on the most severe lesion observed on the section. Final scores for each animal were calculated as a sum of the individual tissue scores for granuloma and AFB in all tissues studied (see Supplementary Material). If a tissue showed TB suspicious lesions but they were not confirmed by culture or ZN that tissue was not included in the final score.

### Statistical Analysis

Data was analyzed both jointly in a first approach and then separately according to the initial TB infection status of each animal. Since most of the considered variables were quantitative or semi-quantitative, the main statistical approaches were submitted to linear models, either analysis of variance (proc GLM) for treatment or correlation (proc corr) for association between quantitative variables. For scores a logarithmic transformation and for proportions an arc sin square root transformation were applied in order to better comply with the condition of normality for this type of analysis. All these analyses were carried out with the SAS statistical package (SAS Inc., NC, USA). For treatment effect comparison, the Tukey-Kramer test for multiple comparisons was applied. Comparison of frequencies for the MRI analysis was carried out with the Fisher exact test of the tables' procedure (proc tables). Standard statistical significance was accepted at *p* = 0.05, however, all actual values are shown.

## Results

### TB Pre-infected Badgers Prior Challenge

**Figure 1:**
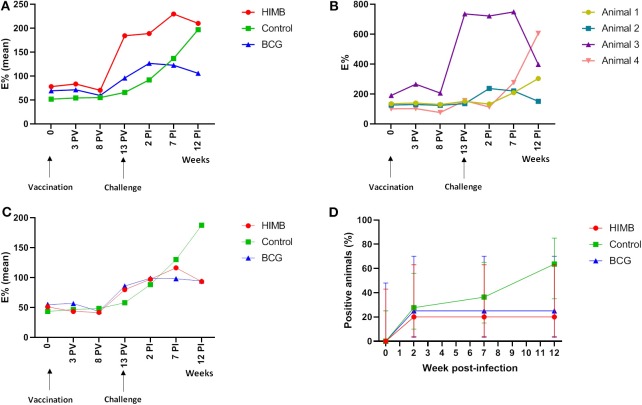
P22 ELISA results throughout the *Mycobacterium bovis* experimental infection in BCG and heat-inactivated *M. bovis* (HIMB) vaccinated animals and controls. **(A)** Mean of E% showed in each group at different time points using all animals (*n* = 24) included in the study design. **(B)** E% displayed by four animals (*n* = 4) that tested positive to P22 ELISA before the challenge. Animal 1 belongs to the control group, animal 2 to BCG group and animals 3 and 4 to the HIMB group. **(C)** Mean of E% showed in each group at different time points when only animals that were negative (*n* = 20) to the P22 ELISA before the challenge were analyzed. **(D)** Percentage (%) of positive animals to the P22 ELISA in different groups using only the animals that showed negative results (*n* = 20) to the ELISA prior the challenge. PV, Post-vaccination; PI, post-infection.

Badgers were tested only by whole blood IGRA ELISA and IGRA ELISPOT before the experiment started and they were all negative. However, as noted below (Table 1, Figure 1), four badgers were positive in the P22 ELISA on the day of challenge which would indicate a previous sensitization to *M. bovis* (TB infection) or other mycobacteria not detected using cellular immune techniques. Sera samples were tested together at the end of the experiment and therefore, we realized that some animals were positive to the ELISA prior challenge when the experiment was finished. That means that we have to take into consideration that experimental results taken on the whole do not adequately represent the protective effects of vaccination. Therefore, serological, MRI, bacteriological and histopathological results will be presented either including all animals or only the negative animals previously to the beginning of the experiment (TB pre-) of each treatment group. The results of the TB positive badgers previously to the beginning of the experiment (TB pre+) do not allow statistical predictions, but are interesting in relation with some variables because they indicate potential consequences of vaccination on already *M. bovis* infected individuals prior vaccination.

**Table 1:** P22 ELISA results in badgers vaccinated with BCG or heat-inactivated *Mycobacterium bovis* (HIMB).

	**Vaccination day-Week 0**	3 weeks PV^*^	**8 weeks PV**	**Challenge**	**2 weeks PI˝**	**7 weeks PI**	**Final day 12 weeks PI**
BCG (*n* = 5)	20% (3.62–62.5)	20% (3.62–62.5)	20% (3.62–62.5)	20% (3.62–62.5)	40% (11.76–76.9)	40% (11.76–76.9)	40% (11.76–76.9)
Control (*n* = 12)	8.3% (1.49–35.4)	8.3% (1.49–35.4)	8.3% (1.49–35.4)	8.3% (1.49–35.4)	33.3% (13.81–60.9)	41.6% (19.33–68.1)	66.6% (39.06–86.1)
HIMB (*n* = 7)	14.3% (2.57–51.3)	14.3% (2.57–51.3)	14.3% (2.57–51.3)	28.6% (8.22–64.1)	28.6% (8.22–64.1)	42.9% (15.82–74.9)	28.6% (8.22–64.1)
TB pre–^**^							
BCG (*n* = 4)	0.0% (0–48.9)	0.0% (0–48.9)	0.0% (0–48.9)	0.0% (0–48.9)	25% (4.56–69.9)	25% (4.56–69.9)	25% (4.56–69.9)
Control (*n* = 11)	0.0% (0–25.9)	0.0% (0–25.9)	0.0% (0–25.9)	0.0% (0–25.9)	27.7% (9.75–56.6)	36.4% (15.17–64.6)	63.6% (35.38–84.8)
HIMB (*n* = 5)	0.0% (0–43.5)	0.0% (0–43.5)	0.0% (0–43.5)	0.0% (0–43.5)	20% (3.62–62.5)	20% (3.62–62.5)	20% (3.62–62.5)

*Percentage (%) of positive animals to the P22 ELISA in the control and vaccine groups*.

* PV, Post-vaccination; ^˝^PI, Post-infection;

** *TB pre-, Results of P22 ELISA using only badgers that tested negative by P22 ELISA prior the challenge. The top half table showed results of P22 ELISA analyzing all animals included in the study*.

*Intervals in parentheses indicate 95% confidence limits*.

### Clinical Signs

All animals (TB pre+ and TB pre-) remained in good clinical condition throughout the study. The mean body weight measured at the time of vaccination was 9.79 kg, SD 1.31 kg; at the time of the experimental challenge 10.94 kg, SD 1.18 kg; and before post-mortem examination 12.14 kg, SD 1.55 kg.

### Cellular and Humoral Immune Response

**Figure 2:**
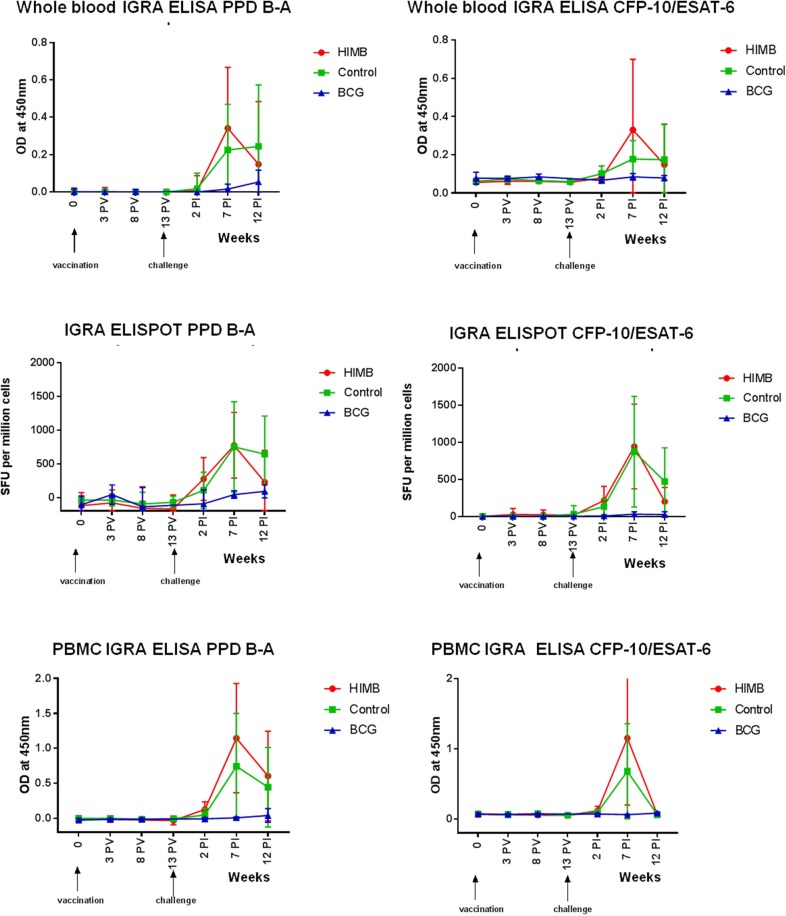
Cellular assays (Whole blood IGRA ELISA, IGRA ELISPOT, and PBMC IGRA ELISA) results throughout the *Mycobacterium bovis* experimental infection in BCG and heat-inactivated *M. bovis* (HIMB) vaccinated animals and controls. All studied badgers (TB pre+ and pre- badgers; *n* = 24) are included in the graphic. PV, Post-vaccination; PI, post-infection.

IFN-γ producing cells responded to combined PPD-B and PPD-A antigenic stimulation in the blood by whole blood IGRA ELISA and IGRA ELISPOT 2 weeks PI peaking at 7 weeks PI (Figure 2). There was a greater response to PPD B-A in control than in BCG vaccinated animals from week 2 PI. However, the PPD B-A response in HIMB animals was greater than in the control 2 weeks PI. The mean peak response to CFP-10 and ESAT-6 was seen 7 weeks PI in the control and in the HIMB vaccinated group. BCG vaccinated animals did not respond to a combination of CFP-10 and ESAT-6 antigens (Figure 2). Ag85A did not add to the overall picture in the IGRA ELISPOT, peaking the response 2 weeks PI in the HIMB group (see Supplementary Material).

The levels of PBMC proliferation (PBMC IGRA ELISA) in combined PPD-B and PPD-A peaked at ~7 weeks PI and persisted for the 12 weeks PI period in the control and HIMB vaccinated badgers (Figure 2). That response was higher in the HIMB group than in the control group. The mean peak response to CFP-10 and ESAT-6 was seen 7 weeks after challenge in the control and in the HIMB vaccinated group (Figure 2). BCG vaccinated animals did not respond to a combination of CFP-10 and ESAT-6 antigens.

One control, one BCG and two HIMB vaccinated animals were positive in the P22 ELISA prior to vaccination and challenge (Table 1), although the antibody levels were low and close to the cut-off in three of them. The number of positive animals rose 2 weeks PI (Figure 1). In BCG and HIMB-vaccinated groups the antibody levels peaked at ~2 weeks PI and only two animals of each group were positive by ELISA at the end of the study. Conversely, eight badgers [66.66% (CI 95% 39.06–86.19)] showed antibodies against P22 antigen in the control group at the end of the study. A significantly higher number of badgers positive by ELISA were observed in the control group compared to vaccinated groups at the end of the study (*p* < 0.001) (Table 1).

### Post-mortem Tuberculous Lesions

**Figure 3:**
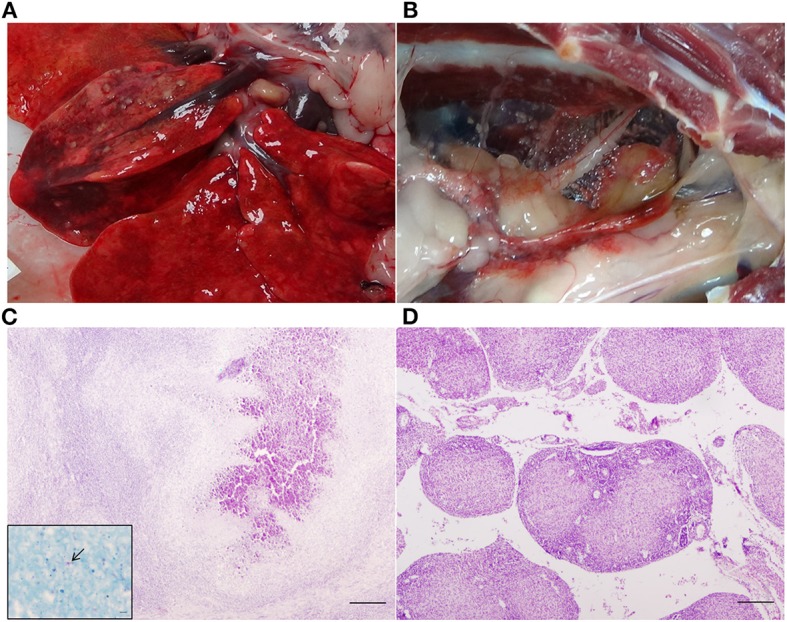
Gross and microscopic lesions in a control badger. **(A)** TB lesions 2 mm in diameter in the right middle lung lobe. **(B)** Miliar TB lesions in the mediastinum. **(C)** Right middle lung lobe: TB granuloma (type 4) in which central mineralization can be observed. Haematoxylin-eosin stain, bar = 200 microns. Inset: Acid fast bacilli (arrow). Ziehl-Neelsen stain, bar = 5 microns. **(D)** Mediastinum: TB granulomas type 2 in which central coagulative necrosis is observed. Haematoxylin-eosin stain, bar = 200 microns.

All except one HIMB-vaccinated badger (that was TB pre-) presented gross TB lesions at necropsy (Figure 3). Badgers exhibited different scores (see Supplementary Material). Comparison of groups (including TB pre+ and TB pre- badgers) showed a statistically significant effect of vaccination (*p* = 0.0048) due to differences between BCG (score 9.0; *p* = 0.081) and HIMB (score 10.3; *p* = 0.0048) with the control, but not between them (*p* = 0.9210).

### Magnetic Resonance Image (MRI) Analysis

**Figure 4:**
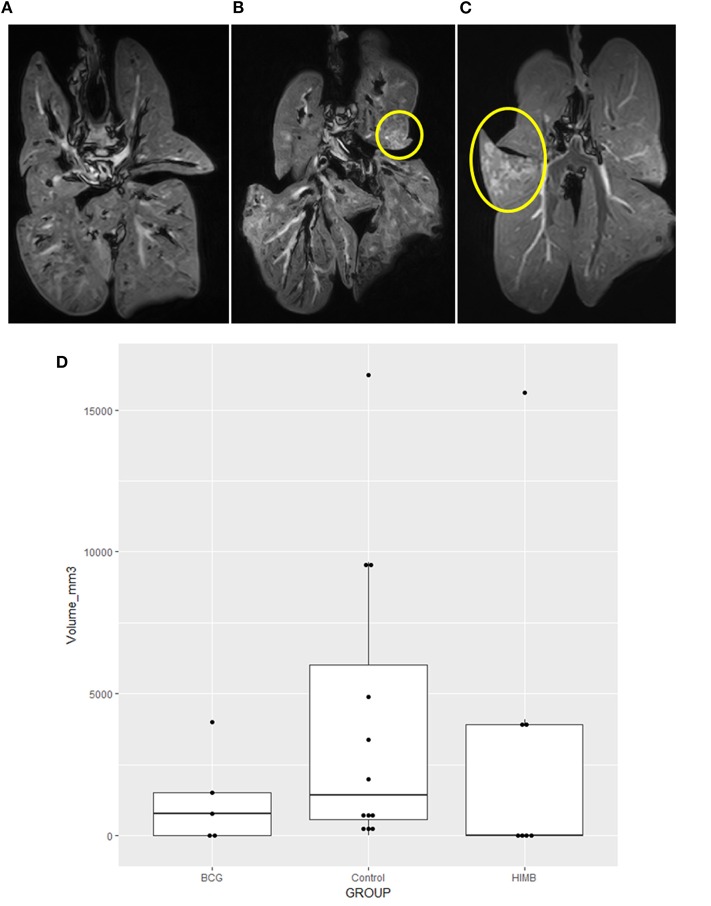
Magnetic Resonance Image (MRI) tuberculosis (TB) lesion volume expressed in volume (mm^3^) in vaccinated and control badgers. Examples of lungs “not infected or with low level infection” **(A)**, “noticeably infected” **(B)**, and “strongly infected” **(C)** with lesions inside the yellow circle. Lower lesion volumes are observed in BCG and heat-inactivated *M. bovis* (HIMB) vaccinated groups compared to the control group **(D)**. Boxes represented the 25 and 75% percentiles; the horizontal lines inside boxes indicate the TB lesion volume (mm^3^) median values. All studied badgers (TB pre+ and pre- badgers; *n* = 24) are included in the graphic.

The MRI analysis showed a broad range of lesion extension in badgers—“strongly infected,” “noticeably infected,” and “not infected or with low level infection”—(Figure 4), whereby volume could be precisely calculated in mm^3^ for each animal (Figure 4). This allowed us to compare TB lesion volumes in BCG and HIMB vaccinated groups with the control group, both quantitatively (mm^3^) and qualitatively (presence/absence). The overall quantitative comparison with the general linear model showed no significant differences between groups (*p* = 0.3057). Restricting the analyses to the volume lesions in TB pre+ and TB pre- animals, differences were not significant in quantitative terms, *p* = 0.8165 and *p* = 0.1481, respectively. However, geometric means in the TB pre- animals showed lower values in TB volume lesions of 98% in the HIMB group (*p* = 0.0548) and of 78% in BCG group (*p* = 0.4540), compared to the control group. This corresponded well with frequency comparisons with the Fisher exact test, *p* = 0.0851 and *p* = 0.2773, respectively, confirming that HIMB (42.9%) animals tended to have less TB lesions than BCG (60%) and control (83.3%) groups.

### Culture, Real Time-Polymerase Chain Reaction (RT-PCR), and Spoligotyping

A total of 527 tissues were individually cultured (see Supplementary Material). The frequency of *M. bovis* isolation was significantly different between groups (BCG: 16.36%; HIMB: 34.42%; Control: 30.80%) (*p* = 0.0038). However, when only the TB- badgers were considered, the vaccinated groups behaved more similarly (BCG: 14.77%; HIMB: 25.45%; Control: 30.99%) (*p* = 0.0122). From a quantitative perspective, the mean log_10_ CFU score from the BCG group (0.49; reduction 50%) was significantly lower than the control (0.98; *p* = 0.0095) and the HIMB (1.25; reduction −28%; *p* = 0.0983), which was nearly significantly larger than the control (*p* = 0.0983). Restricting the analyses to the TB- animals reduced statistical power by half (*R*^2^ = 0.0791 vs. *R*^2^ = 0.1455), but showed that the HIMB group had a lower bacterial burden (0.81; reduction 18%) than the control (0.98) even though this difference was not significant (*p* = 0.3438), while the BCG (0.51; reduction 48%) maintained statistical significance (*p* = 0.0182).

BCG was isolated from the right retropharyngeal LNs of two badgers vaccinated with that vaccine. The remaining spoligotyped isolates from those and the rest of the badgers corresponded with the challenge strain SB0339. *Mycobacterium avium* complex (MAC) mycobacteria were isolated from the right bronchial and posterior mediastinal LNs of two BCG-vaccinated badgers. Additionally, a total of 248 clinical samples (68 tonsillar swabs, 69 nasal swabs, 47 fecal swabs, 20 urine samples, 20 tracheal swabs, and 24 feces) were also individually inoculated. *Mycobacterium bovis* was isolated from one tonsillar swab (from week 7 PI sampling) and one urine (from week 12 PI sampling) obtained from one of the already TB pre+ badger from the HIMB group.

### Histopathology

All badgers (including the badger without visible gross lesions) showed microscopic lesions compatible with TB (see Supplementary Material). If we include all animals (TB pre+ and TB pre- badgers), both BCG (mean 0.48; reduction 28%) and HIMB (mean 0.57; reduction 15%) vaccine groups showed lower mean lesion severity scores but the differences with the controls were not significant (*p* = 0.0980 and *p* = 0.9783, respectively). Consistent with the results for visible lesion scores, BCG and HIMB vaccination reduced the number of sites with histological lesions, as well as the severity of granulomas compared with controls. The mediastinum showed TB microscopic lesions in 10/12 (83.33%) control, 1/5 (20%) BCG, and 3/7 (42.85%) HIMB badgers. Granulomas type 4 (see Methods section) were observed in 10/12 (83.33%) control animals, vs. none for BCG and two (28.57%) for HIMB vaccinated badgers (Figure 3). The right middle lung and the right tracheobronchial and posterior mediastinal LNs contained the most severe lesions. The most frequently affected LN outside of the thoracic cavity was the hepatic LN (18/24, 75%). Mesenteric LN was affected in 4/12 (33.33%) control, 1/5 (20%) BCG, and 2/7 (28.57%) HIMB badgers. Spleen showed TB granulomas in one HIMB and two control badgers. One HIMB badger and two BCG (one of the formers was TB pre+) badgers presented TB granulomas in liver. Kidneys were not affected in BCG badgers, although one control and one TB pre+ HIMB badgers showed lesions in this tissue.

If only TB pre- animals are considered results slightly improved both for BCG (mean 0.47; reduction 31%) and for HIMB (mean 0.54; reduction 21%) again not significantly different from the control group (*p* = 0.1162 and 0.8480, respectively).

### Combined Analyses

#### Serology and Post-mortem Outcome Correlation

There was a significant correlation between serology in the final day and histological final scores (overall *r* = 0.70411, *p* = 0.2959, TB pre+ *r* = 0.93700, *p* = 0.0630; TB pre- *r* = 0.50121, *p* = 0.0244) and MRI volumes (overall *r* = −0.05220, *p* = 0.8130; TB pre+ *r* = 0.93700 0.0630; TB pre- *r* = −0.13034, *p* = 0.5948). Statistically significant correlations were also observed between serology and MRI volumes at weeks 2 (*r* = 0.50975, *p* = 0.0258) and 7 (*r* = 0.47018, *p* = 0.0422) PI.

Serological results had a significant correlation with the total number of colonies isolated from all tissues for the whole set of data (*r* = 0.6562; *p* = 0.0007 for week 0 to *r* = 0.4769; *p* = 0.0128 at the final day of the study), however, this correlation was lost if the data were transformed into the log_10_ score (*r* = 0.2285; *p* = 0.2843 for week 0 to *r* = 0.7317; *p* < 0.0001 at the final day of the study) or TB pre+ were excluded. This was an artifact due to the large number of colonies isolated from the two TB pre+ HIMB vaccinated animals. This correlation is consistent with HIMB vaccination having a negative protective effect on both lesions and bacterial burden, which can be reflected by a quantitative variable like isolation with a huge upper range limit than by a limited range score. This implies it is necessary to separate the TB pre+ and TB pre- animals. In TB pre+ group there was a significant positive correlation between the final day ELISA index and colony log_10_ score (*r* = 0.9710; *p* = 0.0290). The TB pre- group showed a significant positive correlation between the final day ELISA index and both lesion (*r* = 0.5012; *p* = 0.0244) and isolation score (*r* = 0.5088; *p* = 0.0220).

#### Lesion and Isolation

Lesion score and isolation log_10_ score were strongly correlated for the whole set of data (*r* = 0.6886; *p* < 0.0001). Therefore, in order to better synthesize the infection outcome and protection effects, a lesion and bacterial burden index was calculated by addition of both scores (see Supplementary Material). This combined score was smaller than that of the control (1.32) group for the BCG group (0.70; *p* = 0.0046), but larger for the HIMB group (1.46; *p* = 0.4693) that was also larger than the BCG group (*p* = 0.0016). If only the TB pre- animals were taken into account, the combined index for BCG (0.71) and HIMB (1.06) groups did not differ (*p* = 0.1976) between them, and were, respectively, significantly (*p* = 0.0089), and not-significantly (*p* = 0.2161) smaller than the control group (1.33) representing reductions of 46 and 20%.

## Discussion

In this study we report an experimental model of TB in badgers challenged by the endobronchial route with an infective 10^3^ CFU dose of a Spanish *M. bovis* strain. All badgers were successfully infected and developed TB lesions in the respiratory system (100% infection rate), similar to the experiments carried out in the UK and ROI with English and Irish *M. bovis* strains (11, 28). Orally administered BCG and HIMB vaccines reduced values in the severity and volume of gross and microscopic lesions in TB-experimentally infected badgers, and reduced the dissemination of *M. bovis* in vaccinated animals. MRI proved to be a very valuable tool to measure the volume of TB lesions in badger lungs, as shown previously in badgers (Sandrine Lesellier, personal communication) and in other species using computed tomography (i.e., sheep) (25).

Vaccines did not induce full protective immunity, but moderated the severity of the infection although two HIMB-vaccinated animals showed high DBSs (one of them was P22 ELISA-positive prior to challenge). In this sense, we have to take into account the variability between individual animals and between groups of animals, especially when using wildlife species with variable genetic and immunological backgrounds (14). That might have reduced the power to detect a vaccine protective effect, together with the limited number of animals used for this experimental infection. Individual parameters (sex and weight) may affect experimental studies of TB infection in badgers, i.e., female badgers appear more resilient to establishing *M. bovis* infection than male badgers, with longer survival times following the detection of bacterial excretion (29). We did not detect differences between sexes; however the two HIMB-vaccinated animals (one male and one female) with high DBSs were the thinnest in their group which might make them more susceptible to TB infection.

These general conclusions, however need a more detailed discussion because there are several observations that might bring up novel perspectives on modeling TB in badgers and, especially, in the evaluation of protective effects. On one hand it has been seen that cellular immunity alone is not sensitive, as it has been used for selection of wild non-infected badgers, and some of them might not have been so. In experimental studies, IGRA techniques are usually used to confirm the absence of infection before challenge (13, 14). However, fluctuation and variability of IGRA response (30) has been observed in naturally infected badgers. Therefore, if studies only rely on cellular assay screening (i.e., whole blood IGRA ELISA and IGRA ELISPOT), some infected badgers which screen negative via those techniques may in actually be positive via serology. IGRA and ELISA tests in badgers have been used in parallel in experimental and natural infections to detect infected animals and to measure protective immunity in vaccinated populations (13, 14, 31, 32). Serological (P22 ELISA) results presented in this study suggested that some badgers were infected prior to challenge and they were not detected by IGRA techniques. In this regard, in the same way as for diagnosis, whole blood IGRA ELISA, IGRA ELISPOT, and P22 ELISA could be used in parallel to exclude any infected badgers from inclusion in vaccination trials. This is very relevant since our experiment shows very different courses and outcomes depending on the type of vaccine and the badger TB status that we will discuss below. In that sense it is important to point out that this experiment has shown that the P22 ELISA is a powerful tool for preventing the use of already infected animals that can not only yield false protection results, but can also infect in contact animals before the vaccine has induced a protective response. This might even have more theoretical and epidemiological importance since it reveals that cell mediated responses may not detect already infected individuals. Analyzing results separately according to TB status shows that while BCG has a positive effect (reduction in TB scores) on both pre-infected and non pre-infected individuals, the inactivated vaccine has opposite effects depending on TB status before the start of the study. Thus, while HIMB has some protective effect on naïve animals, it seems to induce a worsening effect on already infected ones, both at the pathological and bacterialogical levels. This would be consistent with negative effects reported for other inactivated vaccines as well (33). However, more studies are needed to confirm this hypothesis.

It has been stated that *M. avium* antigen presensitization of vaccinated badgers could potentially negatively impact the response to BCG vaccination and that it might be positively associated with severity of disease (34, 35). We isolated MAC from two BCG-vaccinated badgers (both TB pre- animals) although apparently that did not affect the protection conferred by the vaccine.

Neither BCG nor HIMB produced adverse clinical signs, although BCG was isolated from the retropharyngeal LNs of two badgers, as also observed in previous studies in which BCG was isolated from the same tissues of two badgers (14). This confirms the safety of BCG delivered at high doses by the oral route to badgers, with very limited excretion in feces (36).

Vaccination with BCG and HIMB reduced the extent of seroconversion after challenge, consistent with previous studies with oral BCG in captive badgers (14). One control, one BCG-vaccinated and one HIMB-vaccinated badgers were low level reactors in the P22 ELISA at the moment of challenge, but all had been negative by IGRA prior to the start of the study. No *M. bovis* other than the challenge strain was isolated from those animals, although only three isolates from each animal were spoligotyped and other *M. bovis* strains could have been in a different location. We therefore might conclude that sero-reactivity in those animals was unspecific due to cross-reaction with other environmental antigens. However, another reasonable possibility for these sero-reactivities to P22 could be that these animals had a previous contact or were infected with *M. microti*, since infection with this species is also detectable using P22 ELISA (37). *Mycobacterium microti* isolation by culture, proven to be suitable for other species within the complex, can be quite difficult (37). In addition, if a mixed culture containing both *M. microti* and the challenge *M. bovis* or BCG strains is spoligotyped, the presence of *M. microti* would go unnoticed because the few spacers this species can harbor (if any) are also present in *M. bovis* and BCG strains (38). However, one HIMB-vaccinated badger that presented the highest DBS was high reactive in P22 ELISA prior to challenge, therefore we cannot exclude that it was a true *M. bovis* infected badger. Indeed efficacy of both vaccines improved when those potentially four pre-infected (TB pre+) animals were excluded from the statistical analysis by histology and culture. Outliers with pre-vaccination immune responses have also been observed in other experimental studies in badgers (14). Cellular response to oral BCG in badgers was low probably because mucosal vaccination may stimulate lower peripheral responses and reflect a reduced proportion of the inoculated dose setting established and presented to the immune system (39). The high cellular response in HIMB vaccinated badgers after challenge could be due to a boosting effect (as that vaccine is based on *M. bovis*) which decreased after week 7 PI. The TB pre- group showed a significant positive correlation between the final day ELISA index and both lesion and isolation score that indicated that the P22 ELISA could be a good infection predictor after at least 7 weeks PI.

Among the main *M. bovis* wildlife reservoirs identified, badger and wild boar have been consistently found as relevant actors in the epidemiology of animal TB in multiple countries in Europe. In particular, *M. bovis* infected badgers have been found in Ireland (40), the UK (41), France (42, 43), and Spain (44, 45); infection in wild boar has been described in France and also in Spain where it is considered a reservoir for TB in southern and central regions (5, 42). Measures for preventing the transmission of TB from wildlife to cattle include a combination of farm biosecurity to minimize the interspecies contact, culling to reduce the density of wildlife populations and vaccination of target species (46). In wildlife one requirement for a vaccine is that it should be administered using baits by oral route. At the moment only BCG is allowed for intramuscular administration to badgers in the UK since 2010, but there are limitations for delivery in the field (16). Based on our data, HIMB vaccination appears to be a promising oral vaccine candidate for badgers, pending further development for large scale production, incorporation in baits, minimum protective doses, duration of immunity, safety, and assessment in field conditions. If experimental studies demonstrate that vaccination using baits protects against TB, it would provide a promising and great advance for the delivery of baits in the field in hotspot areas. The advantages of HIMB vaccine with respect to live BCG include: (i) it does not produce adverse reactions in laboratory and farm trials, (ii) there is no risk of vaccine strain survival in vaccinated hosts or in the environment, (iii) there is no risk of sensitizing ruminants to skin test (i.e., give false positive animals when using the test), and (iv) it is stable in storage and in hot climate conditions (17, 47).

## Data Availability Statement

The raw data supporting the conclusions of this article will be made available by the authors, without undue reservation, to any qualified researcher.

## Ethics Statement

The animal study was reviewed and approved by the licensing committees from Government of Principality of Asturias and Government of Basque Country. License reference numbers: PROAE 20/2015 (SERIDA), PROAE 47/2018 (SERIDA), and NEIKEROEBA-2015-009.

## Author Contributions

This study was designed by AB, MB, JP, and RJ, and run by AB, JP, VÁ, SL, DD, FS, IS, JI-L, JG, HA, RJ, and MB. All authors contributed to the manuscript.

### Conflict of Interest

The authors declare that the research was conducted in the absence of any commercial or financial relationships that could be construed as a potential conflict of interest.
