# Supplementary material for: Protective Effect of Oral BCG and Inactivated Mycobacterium bovis Vaccines in European Badgers (Meles meles) Experimentally Infected With M. bovis
Source: Front Vet Sci. 2020 Feb 4;7:41. doi: 10.3389/fvets.2020.00041 (PMC7011093; doi:10.3389/fvets.2020.00041)
Supplement: Supplementary file 1 [file Table_1.DOCX]

Supplementary table. Individual values for ELISA, lesion score, *M. bovis* isolation and combined lesion and isolation variables

|  |  |  | ELISA index | | | | | | | LESION SCORE | | | | ISOLATION | | COMBINED |  |
| --- | --- | --- | --- | --- | --- | --- | --- | --- | --- | --- | --- | --- | --- | --- | --- | --- | --- |
| Treatment | ID | TB status | W0vaccin | W3PV | W08PV | W12chall | W2PI | W7PI | W12PIfinal | MRI | HISTO | GROSS | COLGRAM | | SCO10 | LESION+ISOLATION | |
| Control | 369 | N | 44,5866142 | 52,3903262 | 41,5213723 | 84,2439897 | 80,0516591 | 122,41208 | 78,9077866 | 638 | 12 | 12 | 2388,0585 | | 0,22727273 | 0,71428571 | |
| Control | 1464 | N | 36,6422947 | 40,832396 | 38,9341957 | 42,4200278 | 49,6920326 | 110,172859 | 557,306491 | 573 | 29 | 40 | 1894,08378 | | 0,86363636 | 1,21428571 | |
| Control | 2191 | N | 33,9707537 | 38,484252 | 50,6889764 | 45,3010133 | 50,3477051 | 66,8786012 | 69,188939 | 464 | 18 | 17 | 564,601072 | | 0,18181818 | 2,46428571 | |
| Control | 5338 | N |  | 72,6940382 | 47,3565804 | 56,5865289 | 89,4695013 | 80,1112656 | 64,5609163 | 3388 | 14 | 26 | 12677,7698 | | 0,59090909 | 1,14285714 | |
| Control | 5360 | N | 41,6901012 | 30,2024747 | 29,8945061 | 35,4261872 | 38,8038943 | 63,0041725 | 41,4902233 | 836 | 16 | 25 | 27537,0015 | | 0,40909091 | 0,78571429 | |
| Control | 866 | N | 35,9227291 | 67,1702575 | 70,7041513 | 53,4025223 | 172,236587 | 291,64512 | 248,976753 | 9653 | 15 | 27 | 446,285025 | | 0,40909091 | 0,78571429 | |
| Control | 1500 | + | 134,703384 | 55,6489753 | 129,978981 | 151,308985 | 132,78927 | 209,033613 | 303,616134 | 0 | 14 | 36 | 2000,91025 | | 0,38095238 | 2,25 | |
| Control | 1661 | N | 47,7051651 | 46,0588544 | 50,8933263 | 84,6016292 | 121,687136 | 268,584357 | 286,846811 | 9439 | 15 | 13 | 1581,73878 | | 0,18181818 | 0,85714286 | |
| Control | 3959 | N | 49,2122209 | 44,5743563 | 54,5717288 | 45,9169481 | 91,0633484 | 97,5113122 | 138,187959 | 16230 | 28 | 24 | 3672,15056 | | 0,72727273 | 1,57142857 | |
| Control | 5225 | N | 51,8016167 | 44,548082 | 50,6174461 | 71,8648998 | 71,0245637 | 71,5901745 | 241,565667 | 4885 | 14 | 17 | 1659,95925 | | 0,22727273 | 1,17857143 | |
| Control | 8248 | N | 54,5280175 | 43,6153442 | 56,8838676 | 75,1043115 | 124,886878 | 192,630899 | 180,866283 | 1982 | 20 | 30 | 4471,79534 | | 0,59090909 | 1,71428571 | |
| Control | 9761 | N | 38,1970133 | 32,1728849 | 39,8449816 | 43,0373095 | 80,7530705 | 66,9521655 | 153,328035 | 0 | 13 | 8 | 4109,7229 | | 0,45454545 | 1,14285714 | |
| BCG | 2966 | N | 63,1889764 | 48,2986502 | 35,9227291 | 65,587125 | 58,5535466 | 56,3481025 | 68,8429218 | 772 | 11 | 8 | 98,6277873 | | 0,09090909 | 0,28571429 | |
| BCG | 5179 | N | 43,0399325 | 55,5961755 | 43,1702973 | 115,636797 | 143,214782 | 147,864097 | 49,797524 | 1503 | 12 | 14 | 520,166453 | | 0,18181818 | 0,71428571 | |
| BCG | 5296 | + | 126,012373 | 102,924634 | 89,0669955 | 135,485794 | 237,154778 | 220,226505 | 151,228184 | 0 | 13 | 12 | 62,8372276 | | 0,18181818 | 0,64285714 | |
| BCG | 5770 | N | 51,8419573 | 58,6754781 | 53,9525962 | 93,9996026 | 104,54997 | 109,338367 | 85,0193924 | 4007 | 17 | 13 | 13091,2796 | | 0,40909091 | 1,25 | |
| BCG | 9538 | N | 62,1625422 | 63,8498313 | 40,3616934 | 68,5674548 | 89,4496324 | 78,9588714 | 73,4004397 | 0 | 9 | 2 | 1280,48004 | | 0,18181818 | 0,60714286 | |
| HIMB | 3902 | N | 59,2546924 | 35,6805045 | 55,5701524 | 77,08468 | 105,963154 | 90,1260504 | 56,6342617 | 0 | 4 | 3 | 318,955247 | | 0,13636364 | 0,39285714 | |
| HIMB | 4327 | N | 53,7881902 | 50,8933263 | 37,45402 | 49,9030381 | 96,0245637 | 87,0879121 | 98,2912776 | 0 | 13 | 0 | 1233,94385 | | 0,27272727 | 0,96428571 | |
| HIMB | 7228 | N | 38,0874092 | 47,228061 | 33,4603258 | 100,404008 | 90,6431803 | 117,954105 | 119,925663 | 0 | 30 | 18 | 9519,11923 | | 0,54545455 | 1,92857143 | |
| HIMB | 7603 | N | 45,8692972 | 38,5049921 | 37,2700998 | 58,645766 | 71,7517776 | 76,0180995 | 89,1564318 | 3721 | 12 | 17 | 18681,0291 | | 0,36363636 | 1 | |
| HIMB | 8739 | + | 190,080833 | 265,501839 | 206,607987 | 735,43956 | 721,719457 | 749,305107 | 397,990016 | 0 | 10 | 11 | 90981,96 | | 0,68181818 | 1,46428571 | |
| HIMB | 9413 | N | 58,3367585 | 44,2590646 | 45,0341566 | 112,928248 | 122,026503 | 210,4234 | 103,139779 | 4125 | 11 | 8 | 5515,18156 | | 0,40909091 | 1,03571429 | |
| HIMB | 9980 | + | 100,698726 | 102,430373 | 76,4188124 | 155,446025 | 113,202973 | 276,244344 | 605,359958 | 15610 | 23 | 15 | 57617,0936 | | 1,54545455 | 3,42857143 | |
