# Supplementary figures and images for: Protective Effect of Oral BCG and Inactivated Mycobacterium bovis Vaccines in European Badgers (Meles meles) Experimentally Infected With M. bovis
Source: Front Vet Sci. 2020 Feb 4;7:41. doi: 10.3389/fvets.2020.00041 (PMC7011093; doi:10.3389/fvets.2020.00041)

## Slide 1
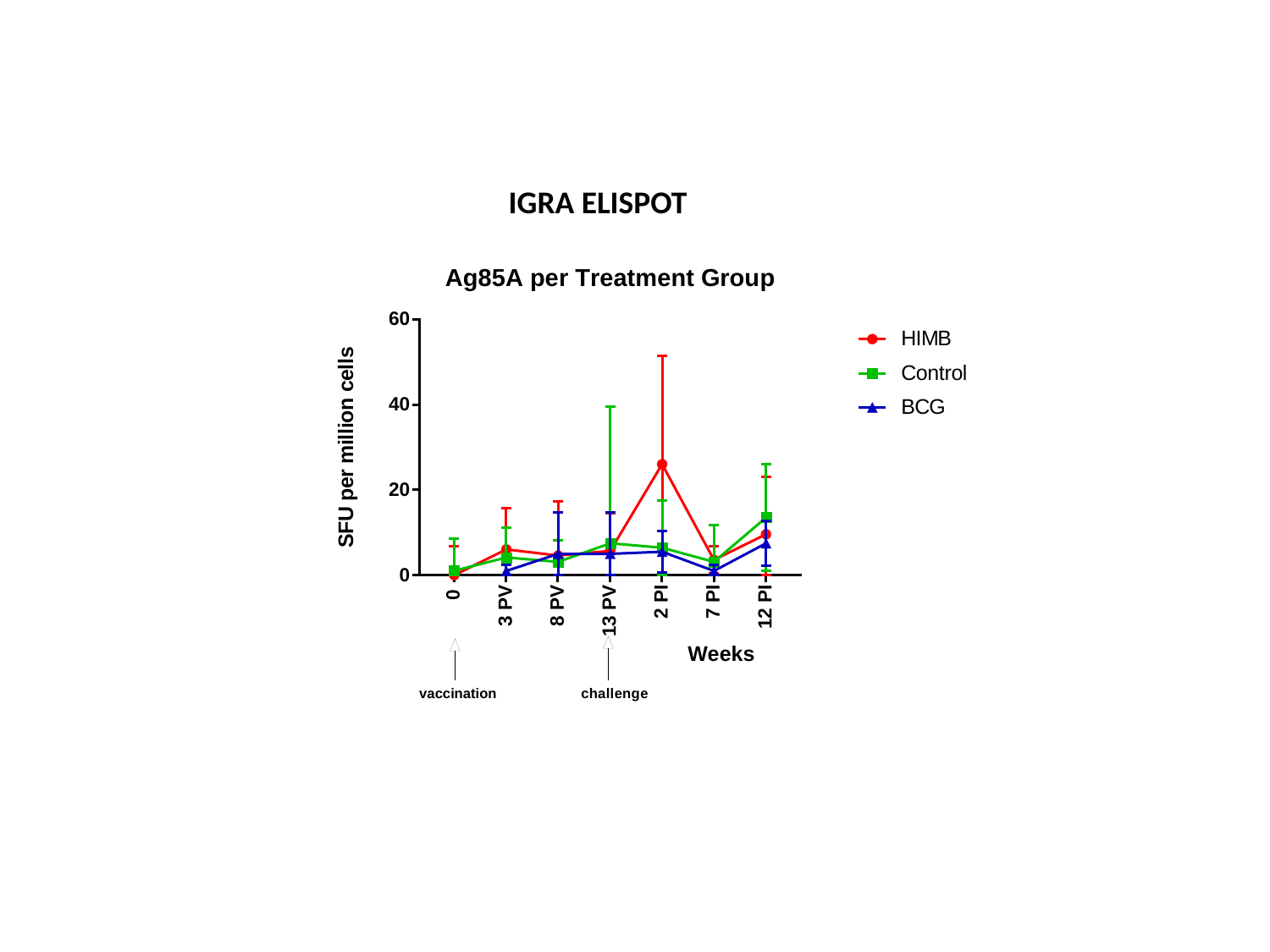

IGRA ELISPOT

Supplement: Supplementary file 6 [file Presentation_1.PPTX]
